# Understanding the lost functionality of ethanol in non-alcoholic beer using sensory evaluation, aroma release and molecular hydrodynamics

**DOI:** 10.1038/s41598-020-77697-5

**Published:** 2020-11-30

**Authors:** Imogen Ramsey, Vlad Dinu, Rob Linforth, Gleb E. Yakubov, Stephen E. Harding, Qian Yang, Rebecca Ford, Ian Fisk

**Affiliations:** 1 Sensory Science Centre, Division of Food, Nutrition and Dietetics, School of Biosciences, University of Nottingham, Sutton Bonington Campus, Loughborough, LE12 5RD UK; 2 Food Flavour Laboratory, Division of Food, Nutrition and Dietetics, School of Biosciences, University of Nottingham, Sutton Bonington Campus, Loughborough, LE12 5RD UK; 3 National Centre for Macromolecular Hydrodynamics, School of Biosciences, University of Nottingham, Sutton Bonington Campus, Loughborough, LE12 5RD UK; 4 Biomaterials Laboratory, School of Biosciences, University of Nottingham, Sutton Bonington Campus, Loughborough, LE12 5RD UK

**Keywords:** Mass spectrometry, Thermodynamics, Fluid dynamics, Molecular biophysics

## Abstract

Consumer sensory evaluation, aroma release analysis and biophysical protein analysis were used to investigate the effect of ethanol on the release and perception of flavour in beer (lager and stout) at different ethanol levels (0 and 5% ABV). Consumer study results showed no significant differences in orthonasal perception, yet retronasal results showed that 0% lager was perceived as maltier with reduced fruitiness, sweetness, fullness/body and alcohol warming sensation (*p* < 0.05). Whilst ethanol alone decreases the aroma release regardless of Log*P*, the presence of α-amylase selectively reduces the headspace concentration of hydrophobic compounds. It was found that ethanol has a subtle inhibitory effect on the binding of hydrophobic compounds to α-amylase, thereby increasing their headspace concentration in the 5% ABV as compared to the 0% beers. This synergistic ethanol * saliva effect is attributed to the changes in the conformation of α-amylase due to ethanol-induced denaturation. It is hypothesised that the partially unfolded protein structures have a lower number of hydrophobic pockets, leading to a lower capacity to entrap hydrophobic aroma compounds. This supports the hypothesis that ethanol * saliva interactions directly impact the sensory and flavour properties of beer, which would provide a basis for further investigations in reformulation of 0% ABV drinks.

## Introduction

Beer is one of the most widely consumed beverages around the world, with production increasing by 3 million litres from 2014 to 2019^1^. However, sales of standard alcohol beer in the UK have been steadily reducing^2^. One of the key factors behind this trend is consumers’ desire to limit their alcohol consumption in order to reduce the risks associated with alcohol-related diseases and other considerations^3^. The worldwide non-alcoholic beer (NAB) market is predicted to increase in value to $25 billion by 2024, as consumers begin to express more interest in lower alcohol counterparts^4^. Therefore, there has been increased development within the NAB sector, with research focusing on understanding the physiochemical properties and sensory attributes of the product matrix in order to improve the quality and experience of the non-alcoholic product.

The composition of the food or beverage plays a key role in the release of flavour compounds^5,6^. These can include the chemical characteristics of volatile compounds (volatility, polarity, and hydrophobicity) as well as the physiochemical properties (chemical composition, physical properties, texture and viscosity). Beer matrix components can be broadly classified into two groups; volatiles which include a wide range of compounds such as aliphatic and aromatic alcohols, esters, acids, carbonyl compounds, terpenes; and non-volatiles which include ethanol and larger macromolecules such as polysaccharides, proteins and nucleic acids, as well as inorganic salts, sugars, amino acids, nucleotides, polyphenols and hop resins^7^. All components found in beer play an important part in the final product matrix, but little is known about the effect these components have on flavour release during consumption. In order to understand the functionality of ethanol, further insights into its’ effects on the organoleptic profile are required to develop low/no alcohol beverages which have the same desirable sensory attributes and high consumer acceptance as standard alcoholic drinks.

To tackle some of the challenges, previous work has looked at the impact of ethanol on the sensory properties of beer. Clark et al.^8^ used a trained sensory panel to identify differences between beers with different ethanol concentrations (0, 2.25 and 4.5% ABV). However, no differences were found in terms of separate aroma and flavour attributes, but an enhanced warming mouthfeel, sweetness and complexity of flavour was observed^8^. In another study Missbach et al.^9^ found that malty was the most pronounced attribute in an alcohol-free beer after swallowing the sample. Perpete and Collin^10^ used purge and trap and thermal desorption cold trap extraction to measure aldehydes in beers with different ethanol concentrations. They found that increasing the ethanol concentration of a beer from 0 to 5% showed increased retention of aldehydes, such as 2-methylbutanal and 3-methylbutanal, which are responsible for the ‘worty’ off-flavours in NAB.

Ethanol clearly has a substantial effect on the overall sensory properties of beer. Consequently, to further scientific understanding of these perceptual changes, researchers have looked at the impact of ethanol on headspace partitioning of volatiles using classical headspace techniques such as solid phase micro extraction gas chromatography mass spectrometry (SPME–GC–MS). These studies mostly found that as ethanol concentration increases there is a decrease in headspace concentration, with ethanol altering the polarity of the product matrix and increasing the solubility of aroma compounds^10–15^. These static headspace techniques are highly useful in the study of aroma interactions within the product, as they can be used to find subtle differences, which may be underestimated by dynamic methods^16^. Though conversely, static headspace measurements alone fail to take into account other conditions such as air sweeping, saliva mixing, mastication and temperature changes, which occur during consumption^17^. To alleviate these shortcomings of static headspace analysis and capture the real life dynamic aspects associated with oral processing, researchers are developing and beginning to apply novel methods.

One of the ways suggested to understand some of the dynamic changes in flavour release is through the analysis of the bolus, which ultimately plays a role in the perception and release of flavour. This is achieved through the inclusion of saliva, or its components, which are known to have a significant effect on the retronasal release through interactions with aroma molecules^18^. Saliva is a complex mixture made up of water (97 wt%) and a range of salivary proteins and electrolytes. Salivary α-amylase, mucins and proline rich proteins (PRP’s) are the most abundant of the salivary proteins, contributing over 90% to the entire salivary protein content^19^. These proteins and glycoproteins are responsible for the key physiochemical properties of the saliva, such as viscoelasticity, lubrication, control of Ca^2+^ super saturation and buffering capacity^20,21^. Past research recognised some of the fundamental roles of saliva, in addition to the ingredients used in the formulation of food. Therefore, scientists began to analyse its effects on the generation of flavour, although studies generally focused on studying the release and partitioning of volatile aroma compounds in single component systems, such as solutions of sugars, salts or individual food proteins^22,23^.

During food oral processing, the interactions between salivary proteins and flavour molecules in the bolus are proposed to have a significant role in flavour perception. Hence, recent studies are now beginning to characterise some of the more complex interactions underpinning the partitioning of aroma compounds from the bolus during the oral processing pathway^24–27^. However, like most studies on food, the majority of research on beer examines the influence of ethanol on the partitioning of individual aroma compounds in water/ethanol solutions^28,29^, although one has examined flavour release in a model beer^17^. Furthermore, to the best of the author’s knowledge no studies to date have investigated flavour interactions during oral processing in a real beer matrix and, in particular, a non-alcoholic one. Addressing this problem is timely due to the rise of NAB sales, and the need for brewers to improve palatability and acceptability in this sector.

Our work aims to support some of these challenges by using a novel combined approach, in order to understand the differences in the physiochemical dynamics of aroma release and flavour perception between a modified commercial 0% and 5% beer, enhanced with the addition of ethanol and a pre-made standard flavour mixture. The influence of product matrix was also explored, through the use of both a lager and stout style beer. The objectives of this study were therefore to explain the orthonasal and retronasal differences in consumer perception of standard and NAB. This was achieved by quantifying the effect of the ethanol * saliva interplay on flavour release through consumer sensory evaluation, headspace analysis of aroma compounds and macromolecular hydrodynamics.

## Results

### Consumer sensory evaluation—orthonasal vs. retronasal aroma release in the perception of lager flavour

**Figure 1:**
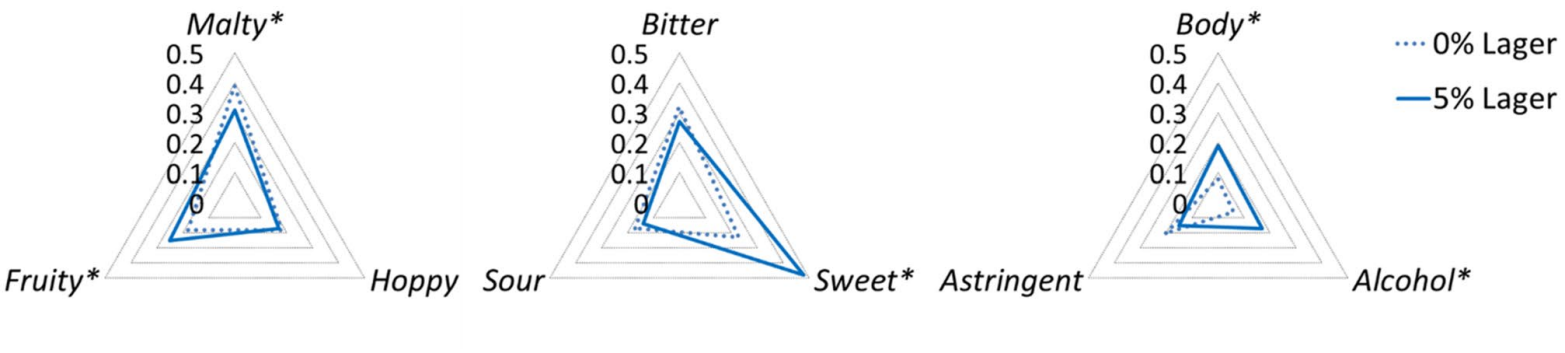
Average proportion of citation for consumer panel using retronasal TCATA sensory attributes divided into flavour, taste and mouthfeel, showing significant differences between samples (Tukey’s HSD Test (p < 0.05)*).

For the consumer analysis, the lager style beer was chosen to understand sensorial differences between the orthonasal and retronasal properties. For the orthonasal analysis, citation rates for the six aroma attributes provided in the lexicon did not reveal any significant differences, apart from minor changes in fruity aroma (Table 1). However, whilst fruity aroma reached significance at *p* = 0.042 it did not show a significant difference in grouping after post-hoc test. In the next part of the study, consumers were asked to consume the lager samples and rate subsequent changes in flavour, taste and mouthfeel attributes over a 60 s time period. This time, the average proportion of citation data from retronasal Temporal Check-All-That-Apply (TCATA) analysis showed significant differences between ethanol samples with respect to flavour, taste and mouthfeel attributes (*p* < 0.05) (Fig. 1). The citation rates for flavour perception of 0% lager were found to be more malty and less fruity, with no significant changes in hoppy flavours. In terms of taste, the 0% lager appeared to be significantly less sweet with no significant changes in bitter and sour attributes. In terms of mouthfeel, significant differences were identified for body and alcohol warming sensation, which scored much lower values for 0% lager (*p* = 0.000 for both attributes). Similar results have been reported previously^8,9,30^, confirming that there are changes in the flavour profile occurring during the short amount of time upon consumption. Therefore, in order to elucidate some of the interaction mechanisms underpinning the perception of flavour in regular 5% ABV beer, the in vitro release of aroma compounds in a 0% and 5% ABV beer was investigated. Here, a stout style beer was also included in order to examine any changes attributed to differences in beer matrix.

**Table 1:** Citation rates of attributes in the description of orthonasal aroma of beer samples.

Aroma attribute	p-value	0%	5%
Fruity	**0.042**	**0.327**	**0.455**
Malty	0.622	0.564	0.535
Hoppy	1.000	0.297	0.297
Stale	0.303	0.356	0.41
Cooked vegetable	0.527	0.475	0.436
Alcohol	0.178	0.218	0.287

Values in bold indicate a significant difference in citation between samples as by Cochran’s Q Analysis and Bonferroni multiple comparisons (p < 0.05).

### Ethanol effect on the release of aroma compounds in different beer styles

**Figure 2:**
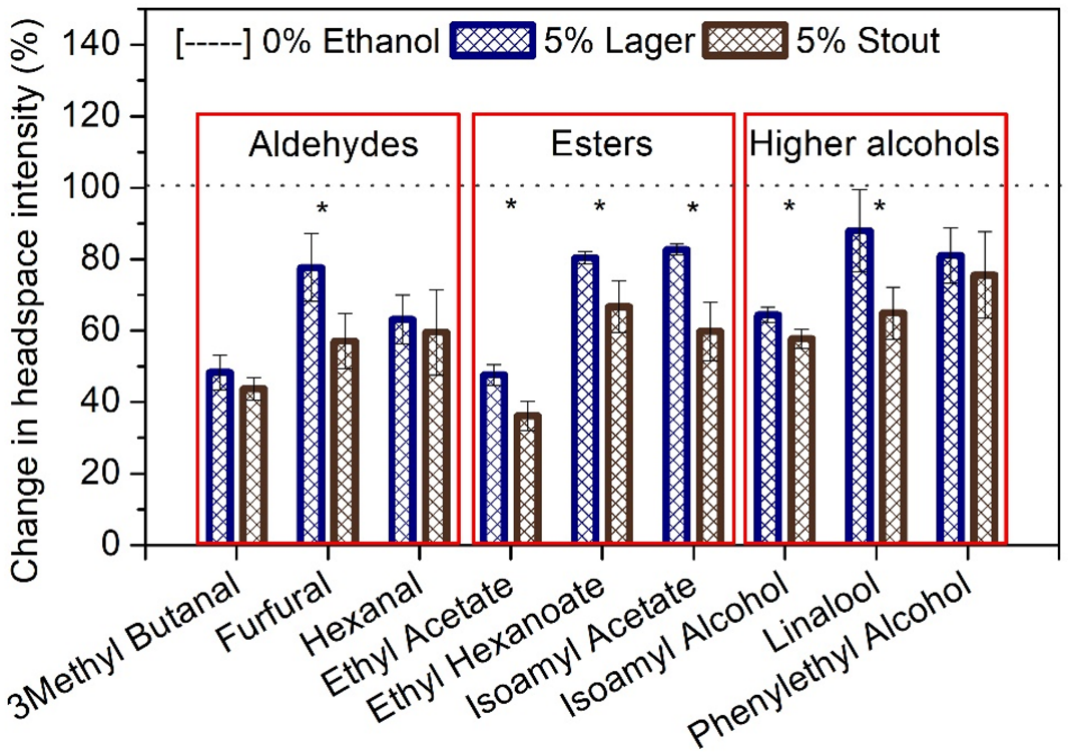
Effect of ethanol on in vitro aroma release (static partitioning) in lager and stout style beers by GC–MS. Data grouped into aldehydes, esters and higher alcohols. Plot shown as relative changes normalised to 0% ethanol (dotted line) for lager and stout (data given as mean ± SE, n = 4). * shows significance (p < 0.05) in volatile release between different beer styles.

Firstly, the effect of ethanol on the partitioning of aroma compounds was examined by GC–MS analysis, in both lager and stout style beers (Fig. 2). Aroma release results were in agreement with the published literature, which revealed significantly lower intensities (*p* < 0.05) in the presence of ethanol (5%) as opposed to the controls (0%), in both beer styles. All compounds except furfural were significantly lower in the presence of ethanol in the lager (*p* < 0.05) although phenylethyl alcohol was not significant in the stout (see supplementary Tab. S2 online). Similar effects of ethanol have been reported when measuring static headspace in model solutions^10,13^. These studies suggested that this is due to ethanol increasing the solubility of aroma compounds in the beer and therefore reducing their partition coefficient and concentration in the headspace^11,14,15^.

Matrix dependant effects were also observed between the two different beer styles; with the aroma concentration in the stout headspace significantly lower than in the lager for most compounds (Fig. 2). This suggests that the flavour matrix interaction is affected by the presence of ethanol and/or ethanol changes the properties of the matrix. In the current study this is attributed to the stout having higher amounts of carbohydrates and proteins present in the sample (6.7 g/100 mL carbohydrates, 3.1 g/100 mL of which sugars, 0.6 g/100 mL protein—information provided on product label), compared to the lager (5.6 g/100 mL carbohydrates, 1.7 g/100 mL of which sugars, 0.3 g/100 mL protein—information provided on product label) suggested to physically lower the release of volatiles in the stout.

### α-Amylase–ethanol interactions in beer

#### GC–MS results

**Figure 3:**
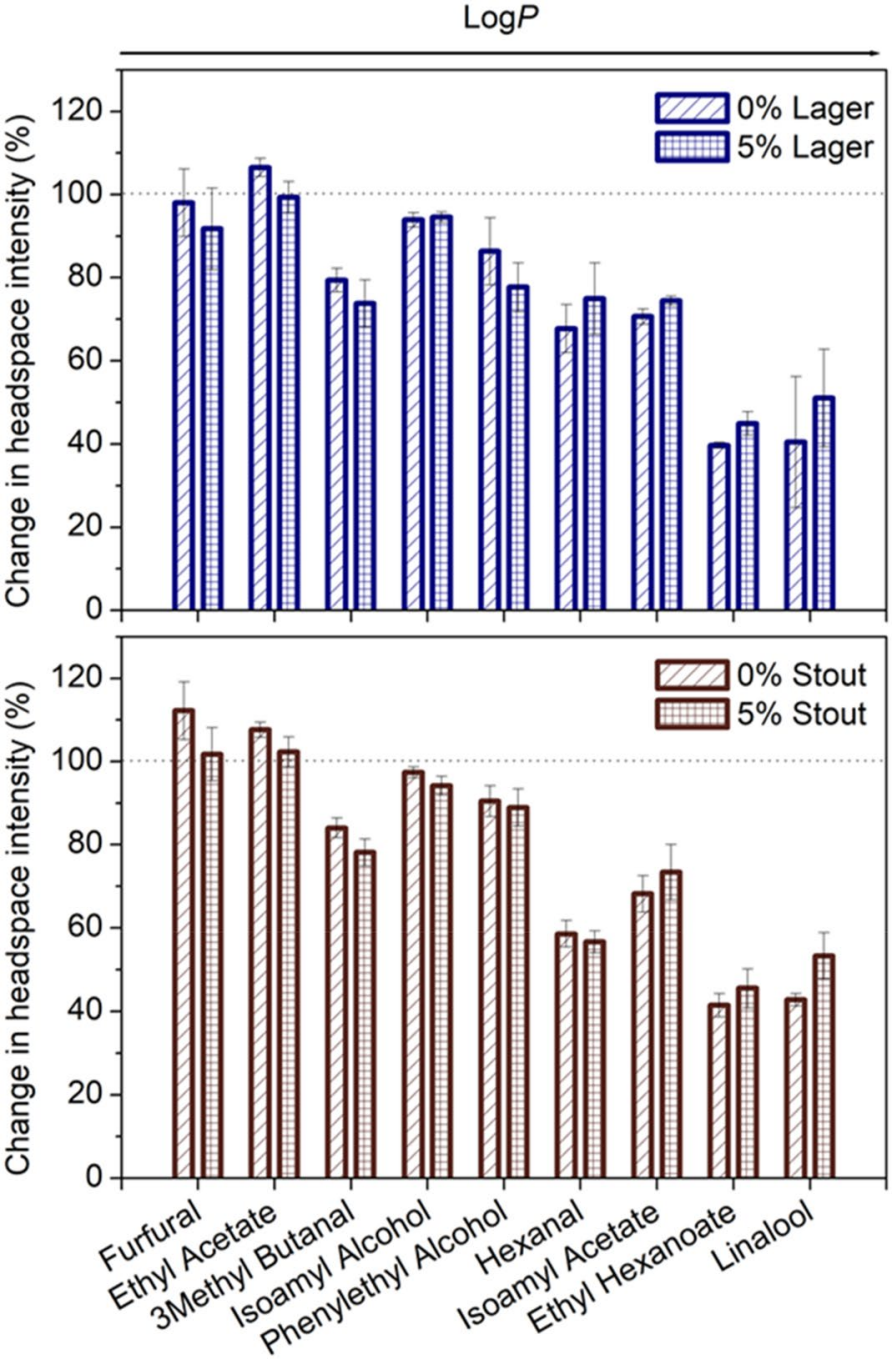
Changes in the static aroma release profile of 0% and 5% beer in the presence of α-amylase by GC–MS. Aroma compounds listed in accordance with LogP coefficient to illustrate the effect of compound hydrophobicity on the aroma-protein interactions. Plot shown as relative changes normalised to controls (respective buffer samples shown as dotted line) for lager and stout (data given as mean ± SE, n = 4).

Salivary α-amylase is the most abundant salivary protein, comprising of over 60% of the total protein concentration in stimulated saliva^31^. To investigate the effect of saliva mixing and bolus formation during oral processing and its effects on the retronasal perception pathway, the effects were evaluated in the presence and absence of α-amylase. It was found that the presence of the salivary enzyme led to a decrease in the aroma release, with significant effects for the more hydrophobic compounds (Fig. 3). Changes are shown relative to their respective controls (buffer samples, before α-amylase addition), corrected for volume to eliminate dilution effect. Of the aroma compounds measured, ethyl acetate, 3-methylbutanal, isoamyl alcohol, hexanal and isoamyl acetate showed significant differences in terms of post-hoc groupings in the lager. Furfural, ethyl acetate, 3-methylbutanal, isoamyl alcohol and isoamyl acetate were significant in the stout (*p* < 0.05) (see supplementary Tab. S2 online).

**Figure 4:**
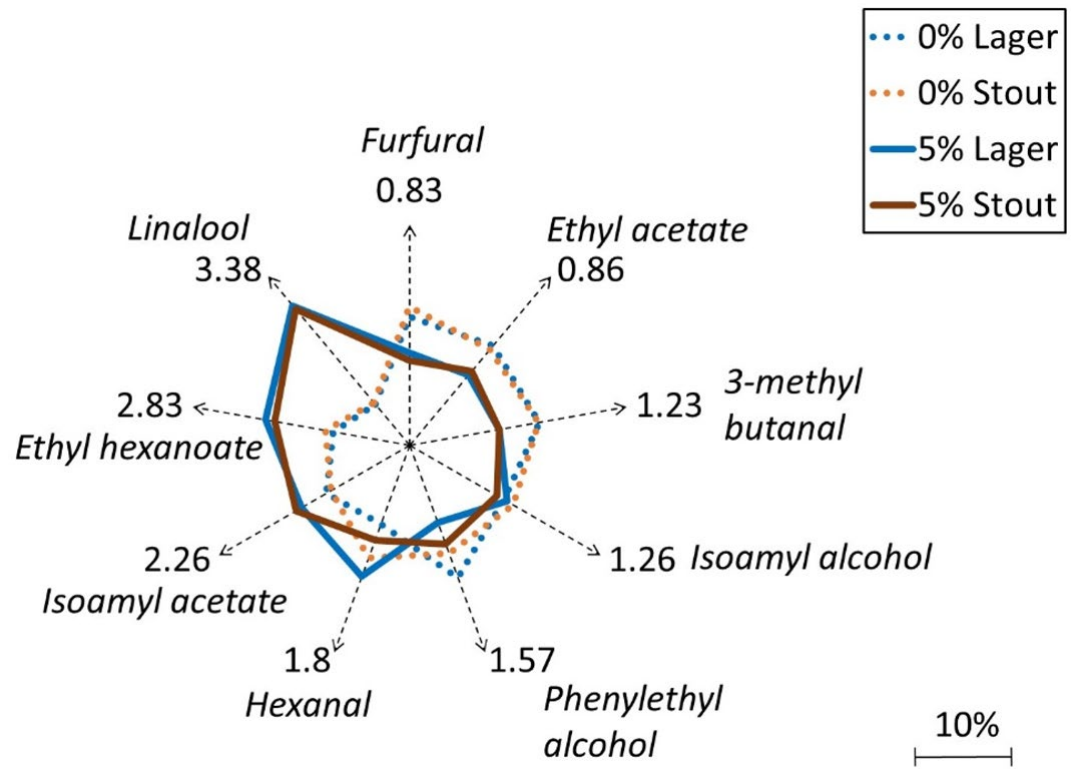
Radar plot analysis of the effect of ethanol on aroma—α-amylase interactions in 0% vs. 5% for different beer styles. Results given as a function of hydrophobicity (log*P*) showing a lower proportion of hydrophilic compounds and higher proportion of hydrophobic aroma compounds released in the 5% lager and stout.

Individual differences in the aroma profile for the lager and stout beers were further analysed in a radar plot as a function of hydrophobicity in order to understand the effect of the salivary protein during the consumption of 0% and 5% ABV beers (Fig. 4). The observed log*P* dependant effects were twofold: the increase of the relative proportion of the hydrophobic aroma compounds in the 5% beers and the decrease of the relative concentration in the 0% beers. Conversely, this meant that the presence of α-amylase led to a higher relative intensity of hydrophilic aroma compounds for the 0% ABV beer, although compounds such as hexanal and phenylethyl alcohol did not appear to follow this trend. A correlation plot is further given as Supplementary Figure S1 online. In addition, this effect was corroborated in both beer styles, acting as a type of validation of the effect helping to provide some clues about the perception differences of NAB, observed via the retronasal evaluation in Fig. 1.

#### Hydrodynamic analysis of α-amylase at different ethanol levels

**Figure 5:**
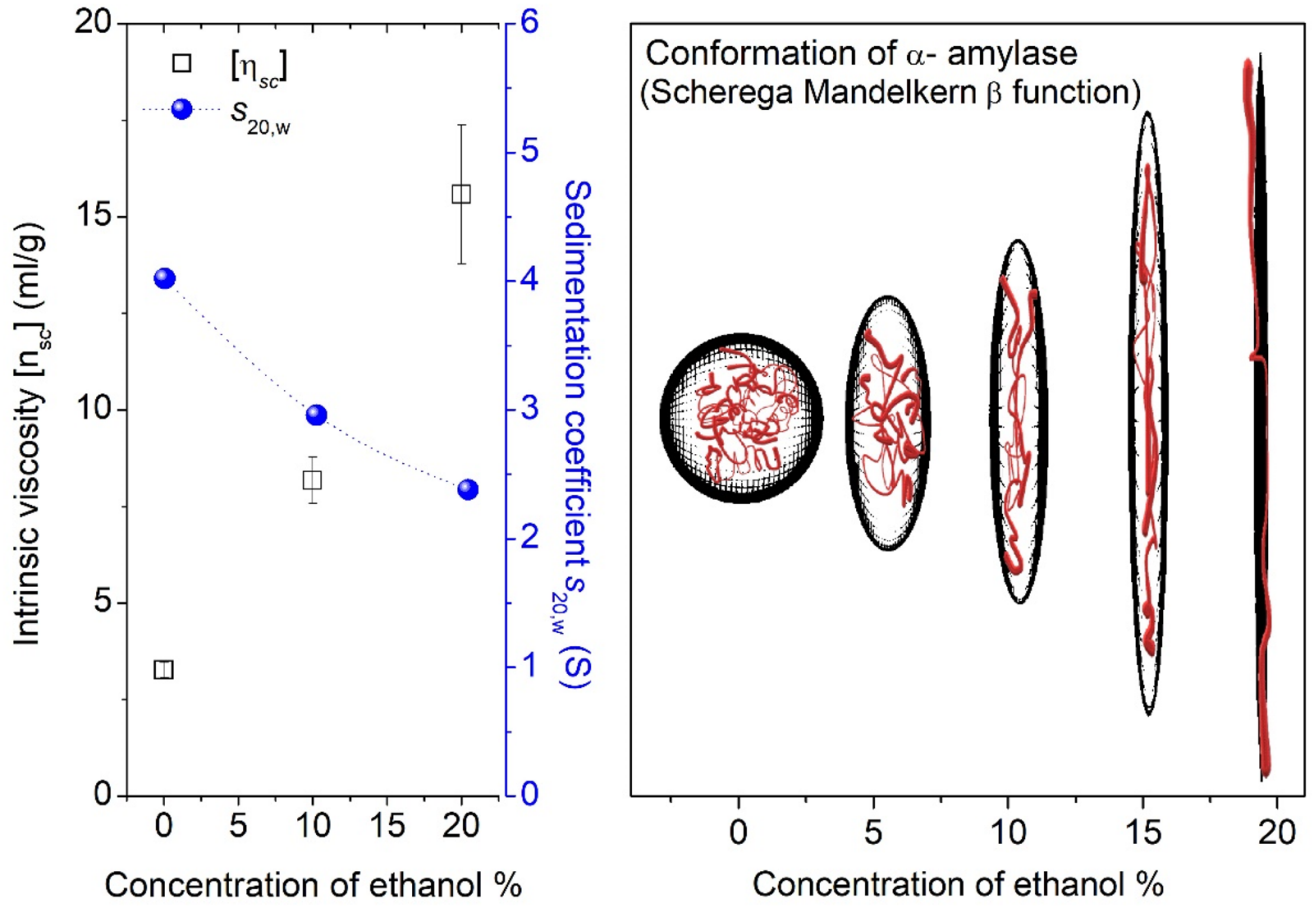
Hydrodynamic analysis of α-amylase as a function of ethanol. Results show the values for the intrinsic viscosity [η_sc_] and sedimentation coefficient s_20,w_ (S), used to illustrate changes in the conformation of α-amylase at higher ethanol concentrations. Prolate ellipsoids were generated in ELLIPS1 using the β-function of the Scherega-Mandelkern equation.

To examine the effects of ethanol on saliva, the hydrodynamic stability of α-amylase was measured in the presence of different concentrations of ethanol. Ethanol was found to have an effect on intrinsic viscosity and sedimentation coefficient of α-amylase. At higher ethanol concentrations, the sedimentation coefficient of α-amylase decreased while the intrinsic viscosity increased (Fig. 5). Combining the two sets of data, a rapid method was employed to determine the gross conformation of the enzyme based on the classical Scherega-Mandelkern relationship^32^. This was achieved by computing the β term in Eq. (2), from the accurate measurements of its hydrodynamic parameters: sedimentation coefficient *s*, intrinsic viscosity [n] and molar mass *M*, by ensuring each series of *s*, [n] and *M* measurements are made in the same ethanol/water solutions. By using the program ELLIPS^33^, the calculated β function values were converted to prolate ellipsoid representations given by their consequent changes in axial ratios (Fig. 5). Since the molar mass of α-amylase is constant, these changes in the anisotropy of α-amylase are suggested to arise from the uncoiling of the polypeptide chain as a result of ethanol denaturation. This effect is essentially a common type of alcohol denaturation where ethanol disrupts the hydrogen bonding of the protein structure, instead forming new hydrogen bonds with the polypeptide chains^34–36^. Although, these effects may differ as a function of protein diversity and heterogeneity in saliva, as well as surface glycosylation, we suggest that the use of α-amylase as a test molecule highlights the generic mechanics and can markedly contribute to the physiological changes, given α-amylase abundance in saliva.

## Discussion

When it comes to analysing the differences in the sensory profile of non-alcoholic beers (NABs), smelling the samples alone (orthonasal evaluation) is not enough to discriminate between aroma attributes, suggesting that ethanol itself has no significant effect on the aroma perception. However, when ingested (retronasal perception), significant differences were determined which showed the 0% beer to be maltier, with reduced fruitiness, sweetness, fullness/body and alcohol warming sensation. This was in full agreement with previous reports for NAB suggesting that saliva is an important factor in sensory perception. This data indicates that product reformulation cannot be based solely on the physiochemical analysis of the product. Similar results were also found by, Peltz and Shellhammer^37^, with ethanol concentration having little effect on the orthonasal detection for specific hop compounds in beer. Missbach et al.^9^ also agrees with these findings, in which they showed that malty is the most pronounced attribute in alcohol-free beer after swallowing. Likewise, others found that NABs have increased aldehyde retention of more hydrophilic compounds such as 2-methyl and 3-methylbutanal and methional, thus increasing worty-off flavours^10^. The same effect was shown in the headspace and sensory analysis, although consumers were given the attribute ‘malty’ to signify this change. Ethanol has also been found to enhance sweetness, alcohol warming sensation^8^ and fullness/body^30^ confirming the results found in this study. Therefore, it was indicated that ethanol has a significant effect on the retronasal perception of beer. Other reasons for the differences in non-volatile attributes could also be explained by multimodal flavour perception, as ethanol is perceived by gustatory, olfactory and trigeminal modalities^38^. However, volatile related attributes appear to be down to the interactions with salivary proteins, which has been suggested in previous research on bioactive food ingredients^25–27^.

In order to examine the ethanol * saliva hypothesis in more detail and provide a mechanism-based understanding, a series of in vitro experiments were designed to evaluate the effect of ethanol, beer matrix and effect of salivary proteins, which are discussed further. Key aroma compounds that impart the recognised and desirable flavour of beer (aldehydes, esters and higher alcohols^39^) were chosen to understand differences in the aroma release of beer. At 5% ABV, the headspace intensity of aroma compounds was lower than in the 0% ABV for both beer styles due to the solubility of aroma compounds in ethanol, reducing their concentration in the headspace^11,14,15^. All compounds were affected in a similar way by the presence of ethanol, and the rate at which they were released could not be explained by their physiochemical properties. Previous research has shown that hydrophobicity plays a role, with more hydrophobic compounds showing a significant decrease in headspace concentration with increasing ethanol concentration^11,40^. However, both of these studies used APCI-MS with model solutions at much higher alcohol concentrations, therefore this theory may not apply to a complex matrix system such as beer.

The effect of product matrix was analysed by comparing the aroma release from lager and stout, the latter having a higher macromolecular content. As a result, aroma release in the stout was lower in comparison to the lager. Previous research by Castro and Ross^7^ has shown that the non-volatile matrix affects the headspace partitioning, as well as the sensory perception of volatile compounds in a model beer due to a physical suppression effect. Other research has also shown that an increased proportion of macromolecules in solution affects the rate of diffusion of aroma compounds, thereby leading to a lower aroma release^41–44^.

The presence of α-amylase had a significant effect on the perception of flavour in both beer styles with the GC–MS analysis showing that the rate at which these compounds change is dependent on compound hydrophobicity, especially pronounced for higher log*P* compounds such as ethyl hexanoate and linalool. It is suggested that this effect is due to hydrophobic interactions between α-amylase and the aroma compounds. Previous research has confirmed these types of hydrophobic interactions, with an increase in the retention of aroma compounds by components found in saliva (mucin and α-amylase)^45–47^ as these aroma compounds are known to bind to salivary proteins and other macromolecules. Muñoz-González et al.^48^ also found that the oral release of ethyl hexanoate and isoamyl acetate was not affected by variations in ethanol content in wine directly. These researchers used an intra-oral SPME procedure where they captured volatiles on a SPME fibre immediately after panellists had rinsed and expectorated wine samples^48^. This consequently shows the impact of using in-vivo or ex-vivo techniques that factor in more real-world consumption dynamics, such as interaction with saliva to form a bolus and its subsequent effects on taste and aroma release.

Although a remarkable effect, the effect of changing from hydrophilic malty to hydrophobic fruity flavours with the addition of ethanol is not a new finding, which has been confirmed by previous research by Boothroyd et al.^40^. They observed that during the dilution of spirits to lower ABVs for nosing, some molecules are more likely to go through structural changes and form agglomerates, which capture hydrophobic aroma compounds. They discussed that this lowers their release into the headspace and changes the aroma of lower ethanol content solutions towards more polar, hydrophilic compounds. Current findings are conceptually similar to some observations reported in the previous work^40^, but in addition they provide a deeper insight into the role of salivary proteins, subjected to a certain degree of ethanol denaturation. This hypothesis was probed through molecular hydrodynamics by analysing the anisotropy of the enzyme, in the presence of different ethanol concentrations. Results found that higher ethanol concentrations increased intrinsic viscosity and decreased sedimentation coefficient. Through computational analysis, it was shown that the conformation of α-amylase changed from globular to elongated structures, suggested to arise from the uncoiling of the polypeptide chain as a result of ethanol denaturation. This common type of alcohol denaturation disrupts the hydrogen bonds of the globular protein structure, whilst instead forming new hydrogen bonds between its polypeptide chains^34–36^. In terms of the mechanism of interaction with aroma compounds, this corresponds directly to a decrease in hydrophobic pockets, which correlates with the shift in the intensity to more hydrophobic aroma compounds in the 5% ABV beers. These changes in the hydrodynamic properties of salivary proteins, including higher viscosity and changes in conformation are suggested to be strongly correlated with the changes in the sensorial perception of beer, including flavour and mouthfeel effects confirmed through the retronasal evaluation. Similar changes in the hydrodynamic properties of salivary proteins are suggested to be responsible for a specific flavour profile i.e. more fruity/estery hydrophobic compounds such as linalool, ethyl hexanoate and isoamyl acetate in the 5% ABV. Conversely, more worty/malty compounds such as the more hydrophilic furfural and 3-methylbutanal appeared to be more enhanced in the absence of ethanol, in both beer styles.

Together, these findings illustrate the importance of linking sensory data with analytical techniques in order to enhance the current understanding of physiochemical changes occurring during food and beverage oral processing, also highlighted in Ickes and Cadwallader^49^. In particular, the combined approach is instrumental for the analysis of intra-oral interactions, which offers brewers a new opportunity for matrix design with controlled oral processing characteristics, flavour release and perception of beer. For NABs, the understanding of the dynamics of flavour release is particularly important for replacing the lost functionality of ethanol and unlocking new dimensions in formulation design. It was suggested that some of the lost functionality of ethanol may be tackled by the addition of dextrins or glycerol which can act as ‘ethanol-mimics’ and help increase aldehyde retention^10^. Further research into oral mucoadhesive might become an attractive option in beer reformulation, by modulating an increase in the retention of more hydrophobic compounds^50^. As observed in Dinu et al.^50^ the development of oral mucoadhesives can lead to a decrease in the interactions of aroma compounds with α-amylase. Balancing these effects could provide brewers with significant guidance on the development of a NAB base recipe, in order to reduce the effects of beer dealcoholisation.

## Concluding remarks and future work

In an attempt to provide an integrated approach in evaluating perceptual and physical changes during consumption of 0% ABV beverages, this study used consumer sensory evaluation, GC–MS aroma analysis and hydrodynamic protein analysis. The aim was to understand the impact of ethanol (0 and 5% ABV), saliva and their interactions on the flavour of two different beer styles: lager and stout. Firstly, consumer sensory evaluation demonstrated that orthonasal perception of aroma alone is not enough to allow significant discrimination between the 0% and 5% lagers. However, in the retronasal TCATA analysis, discrimination of flavour, taste and mouthfeel attributes in 0% and 5% beer was possible. This confirmed the ethanol * saliva interaction effect and provided key evidence that this complex interaction can affect the sensory attributes of lager. The phenomenon appeared to influence the flavour profile of 0% ABV beer, which shifted to more hydrophilic molecules, while the 5% ABV samples had a higher relative proportion of more hydrophobic compounds. This effect was observed in both lager and stout beer types and was linked to ethanol denaturation of salivary proteins, resulting in an extended polypeptide which has fewer hydrophobic pockets that can trap aroma molecules. Further mechanistic investigations are suggested, particularly using other key components in our saliva such as mucins, PRP’s and other glycoforms of α-amylase.

## Materials and methods

### Consumer sensory analysis

#### Participants

To assess the influence of ethanol on perception of beer, 101 consumers (53 men, 48 women), who self-reported consumption of beer at least once every two months, were recruited to take part. Ages ranged from 19 to 70 years of age, with a mean age of 32. Approval from the University of Nottingham Medical Ethics Committee (G10022017) was granted before the study commenced and research was performed in accordance with the Institute of Food Science and Technology Guidelines for Ethical and Professional Practices for the Sensory Analysis of Foods. All participants gave written informed consent to participate in the study and were offered an inconvenience allowance for their time.

#### Samples

A 0% ABV lager (Carlsberg, Northampton, UK) was used as base beer from which two experimental beer samples (0 and 5% ethanol) were prepared. To create the 5% ethanol beer samples, 30 mL of ethanol water mixture (18.09 mL of 96% food grade ethanol (VWR International, Lutterworth, UK) and 11.91 mL of water (Danone, Paris, France) was added to 300 mL of commercial beer. To create the 0% ethanol beer samples, 30 mL of water was added to ensure that all samples had the same concentration of matrix components. On the day of testing, 30 mL of beer was removed from a 330 mL commercial bottle, and the desired ethanol/water solution was added back, with inversion of the bottle to ensure adequate mixing. A lager style beer was chosen for this part of the study, as this is the beer style with the largest market and so there is a larger commercial relevance. For evaluation by consumers, 30 mL of beer was poured into plastic serving cups and served, with each bottle prepared serving no more than 10 consumers. This method was used to minimise sample handling and limit the decarbonation and volatilisation of the samples.

#### Procedure

Consumers participated in the study at the Sensory Science Centre, Sutton Bonington Campus, University of Nottingham, with tests performed at room temperature in an air-conditioned room, under Northern Hemisphere daylight and in individual booths, which conform to ISO standards (ISO 8589: 2007). Data was collected using Compusense software (Guelph, Ontario, Canada).

The session started in a discussion room, where a familiarisation task (15 min) took place. Previous research has shown that familiarising consumers with the methods used to assess products can result in an increase in the ability of consumers to discriminate amongst samples^51^. Consumers were also familiarised with the attributes and definitions they would be using (shown in Supplementary, Table 1). Further details on attribute generation are discussed in Check-all-that-apply (CATA)—orthonasal pathway section. Consumers then evaluated samples in isolated sensory booths (45 min). Check-All-That-Apply (CATA) was used to assess orthonasal aroma attributes and Temporal Check-All-That-Apply (TCATA) was used for in-mouth retronasal sensory attributes, including taste, flavour, mouthfeel and aftertaste.

Beer samples (n = 2) were presented monadically under Northern hemisphere lighting, in a randomised order, according to a Williams Latin Square Design^52^. The attribute order was also randomised across subjects to balance bias associated with list order for both CATA and TCATA attributes. The attribute list order was consistent for a given panellist across all samples^53^. Data were captured using Compusense Cloud software (Guelph, Ontario, Canada). To minimise fatigue and carryover, consumers were given a forced 2 min break between each sample, and were told to take at least 2 sips of water during this break to cleanse the palate.

#### Check-all-that-apply (CATA)—orthonasal pathway

Consumers were asked to assess the presence of six aroma attributes within each sample with the use of a predefined CATA checklist. The attribute list and definitions were generated after a pilot study with six naïve beer consumers (see Supplementary Tab. 1 online). Consumers were advised to take 2–3 short sharp sniffs of the sample and then a longer sniff before clicking on the attributes they perceived.

#### Temporal check-all-that-apply (TCATA)—retronasal pathway

Consumers were then asked to assess the presence of 10 predefined attributes within each sample using TCATA, which is a developed sensory method focusing on all attributes, not just dominant, in the sample over time. This method was chosen for retronasal attributes such as flavour, taste and mouthfeel as beer has a complex profile which changes over consumption time. Ten attributes were selected so as not to exceed the recommended maximum for consumers^54^. Attributes and definitions were developed in reference to published literature^55–58^. Prior to the test, consumers were instructed to familiarise themselves with the position of the attributes on screen, which were presented in a three-column format.

### Physiochemical analysis

#### Samples

A 0% ABV lager (Carlsberg, Northampton, UK) and a 0% ABV stout (Big Drop Brewing Co, Ipswich, UK) were used as base beers and were selected due to following the same NAB production method (altered brewing parameters). Two experimental beer samples (0 and 5% ethanol) were prepared for each beer style, as given in the consumer sensory analysis sample preparation section. These samples were then spiked with a pre-made standard flavour mixture for GC–MS measurements in order to achieve adequate signal, due to being diluted with either water or α-amylase. The volatile compounds used included: aldehydes (3-methyl butanal, furfural and hexanal), esters (ethyl acetate, ethyl hexanoate and isoamyl acetate) and alcohols (isoamyl alcohol, linalool and phenylethyl alcohol) (Sigma Aldrich, Dorset, UK) selected due to their contribution to beer flavour, as well as differences in chemical properties. A stock solution of these compounds was made in 95% ethanol and this was then transferred into the ethanol/water mixtures to ensure consistency. Final concentrations within the modified beers were as follows: ethyl acetate (8.44 mg/L), isoamyl acetate (0.40 mg/L), ethyl hexanoate (0.41 mg/L), isoamyl alcohol (40.78 mg/L) phenylethyl alcohol (9.96 mg/L), hexanal (0.81 mg/L), furfural (5.99 mg/L), 3-methyl butanal (4.07 mg/L) and linalool (0.92 mg/L). These concentrations are typically found in lager beer for these compounds^39^. Physiochemical characteristics for all of these compounds can be found in Table 2. Samples were stored at 4 ± 2 °C prior to sampling.

**Table 2:** Hydrophobicity of flavour compounds (log*P*) and their sensory descriptors (Flavournet, 2004).

Volatile compound	Log P	Flavour in beer
Furfural	0.83	Bread, almond, sweet
Ethyl acetate	0.86	Solvent, fruity, pineapple
3-Methyl butanal	1.23	Malt
Isoamyl alcohol	1.26	Whiskey, malt, burnt
Phenylethyl alcohol	1.57	Honey, spice, rose, lilac
Hexanal	1.80	Grass, tallow, fat
Isoamyl acetate	2.26	Banana, apple, solvent
Ethyl hexanoate	2.83	Apple peel, fruit
Linalool	3.38	Flower, lavender

#### α*-Amylase solution preparation*

The α-amylase solution was made by preparing 10 mg/mL α-amylase from *Bacillus licheniformis* (Sigma A4551) in 0.1 M phosphate buffered saline (Sigma Aldrich, Dorset, UK)^59^. The concentration of buffer and amylase were chosen to mimic the concentration of salivary α-amylase and electrolytes in saliva^31^.

### Gas chromatography analysis

To detect volatile compounds, Solid Phase Microextraction Gas Chromatography Mass Spectrometry (SPME–GC–MS) was used. Beer samples (2 mL) and either buffer or α-amylase solution (2 mL) were transferred into glass vials at a 1:1 ratio. Analysis of volatile aroma compounds was performed using a Trace 1300 series Gas Chromatograph coupled with a single-quadrupole mass spectrometer (Thermo Fisher Scientific, Hemel Hempstead, UK). The method used was modified from Yang et al.^60^. Briefly, samples were incubated at 40 °C for 2 min with shaking. A 50/30 μm SPME Fibre (DVB/Carboxen/PDMS StableFlex, Supelco, Sigma Aldrich, UK) was used to extract volatile aroma compounds from the sample headspace (extraction for 10 min then desorption for 1 min). The injector temperature was set at 200 °C in splitless mode (constant carrier pressure 18 psi (124 kPa). Separation was carried out on a ZB-Wax capillary GC column (30 m × 0.25 ID; Phenomenex Inc, Cheshire, UK). Column temperature was held initially at 40 °C for 2 min, increased by 8 °C/min to reach 240 °C and held for 1 min. Full scan mode was used to detect volatile compounds (mass range from m/z 35 to 200). Volatile compounds were identified by comparison of each mass spectrum with either the spectra from standards analysed in the laboratory or with spectra in reference collections (NIST Mass Spectral laboratory).

### Sedimentation velocity-analytical ultracentrifugation

The effect of ethanol on sedimentation velocity of α-amylase was examined using the Optima XL-I analytical ultracentrifuge (Beckman, Palo Alto, USA) equipped with Rayleigh interference optics. For the sedimentation experiments, 395 μL and 405 μL aliquots of solution and solvent, respectively, were injected into the 12 mm double sector epoxy cells with sapphire windows and run at 40,000 rpm (120,000 g) at 20 °C. The results were analysed in SEDFIT using the c(s) processing methods by generating sedimentation coefficient distributions, s_20,w_ (in Svedberg units, S = 10^–13^ s) normalised to standard conditions (viscosity and density of each solvent at 20 °C).

### Ostwald capillary viscometer

Flow times of the respective ethanol/water solvents (t_0_) and α-amylase solutions (t_s_) were measured using the semi-automated (Schott Geräte, Hofheim, Germany) U-tube Ostwald capillary viscometer immersed in a temperature controlled water bath at 20 °C. A constant volume of 2 mL was sampled at constant α-amylase concentration of 10 mg/mL. The intrinsic viscosity, [η] was calculated according to the Solomon-Ciuta Eq. (1)^61^:

1 $${\left[\eta \right]\stackrel{\sim }=\frac{1}{c}\left(2\left({\eta }_{sp}\right)-2ln\left({\eta }_{r}\right)\right)}^{1/2}$$

### Data analysis

#### Consumer data: CATA and TCATA

##### CATA

Analysis of CATA data followed previous work by Meyners et al.^52^. This was performed by counting the number of assessors that checked each given attribute, forming a contingency table. Cochran’s Q analysis with Bonferroni as a multiple comparison was then performed to show significant differences among samples for each aroma term.

##### TCATA

The analysis of the average proportion of citations followed a similar method as McMahon et al.^57^, with each attribute being assessed as the proportion of the 60 s time period in which it was selected. For example, if malty was checked for a duration of 15 s and hoppy for 25 s, the proportion of citations for malty would be 15/60 = 0.25 and for hoppy would be 25/60 = 0.42. A two factor ANOVA (sample, panellist) and Tukey’s HSD post hoc test was then performed to understand the significance of each attribute.

### GC–MS

To calculate the separate effect of ethanol and α-amylase interactions with beer, all GC–MS samples were analysed in 4 replicates, using a one-way analysis of variance (ANOVA) and Tukey’s post hoc test to identify significance (*p* < 0.05). The percentage changes were then calculated, relative to their controls. For instance, for the effect of ethanol, the 0% samples were considered controls and for the effect of saliva, the water samples were controls. To quantify the effect of α-amylase interactions with different ethanol beers, a two-way ANOVA with Tukey’s post hoc test was performed to understand the interactions of ethanol and saliva on the two different beer styles, with Pearson’s correlation coefficient calculated to construct a correlation map to understand the relationship between factors.

### Hydrodynamics

The theory of Scheraga and Mandelkern^32^ was applied to evaluate molar mass using experimentally determined sedimentation coefficient distribution and intrinsic viscosity. The model assumes that a macromolecule can be represented by an ellipsoidal shape, using the following Eq. (2):

2 $$M={\left(\frac{{N}_{\mathrm{A}}{s}_{20,w}^{^\circ }[{\upeta ]}^{1/3}{\eta }_{o}}{\upbeta (1-\stackrel{-}{} {\uprho }_{o})10{0}^{1/3}}\right)}^{3/2}$$

where *M* is molar mass (g/mol), *N*_A_ is Avogadro’s constant (mol^−1^), [η] is the intrinsic viscosity, η_o_ is solvent viscosity, s°_20,w_ is sedimentation coefficient distribution, v̄ is the partial specific volume of the protein, ρ_o_ is the density of the solvent (g/cm^3^) and β is a shape function, ranging from 2.11 × 10^6^ for spheres to 2.55 × 10^6^ for elongated molecules. As the molecular weight of α-amylase is known, the formula was rearranged in order to obtain the shape function β, which is used for the determination of the axial ratio of a prolate ellipsoid in the program ELLIPS 1^33^.

## Supplementary information

Supplementary Information.
