# Supplementary material for: Understanding the lost functionality of ethanol in non-alcoholic beer using sensory evaluation, aroma release and molecular hydrodynamics
Source: Sci Rep. 2020 Nov 30;10:20855. doi: 10.1038/s41598-020-77697-5 (PMC7704625; doi:10.1038/s41598-020-77697-5)
Supplement: Supplementary file 1 — Supplementary Information. [file 41598_2020_77697_MOESM1_ESM.docx]

**Supplementary Materials**

Understanding the lost functionality of ethanol in non-alcoholic beer using sensory evaluation, aroma release and molecular hydrodynamics

Imogen RAMSEYᵃᵇ, Vlad DINUᵇᶜ, Rob LINFORTH^b^, Gleb E. YAKUBOV^cd^, Stephen E. HARDING^c^, Qian YANGª, Rebecca FORDᵃ and Ian FISKᵇ*

*ªSensory Science Centre, Division of Food, Nutrition and Dietetics, School of Biosciences, University of Nottingham, Sutton Bonington Campus, Loughborough LE12 5RD, United Kingdom*

*ᵇFood Flavour Laboratory, Division of Food, Nutrition and Dietetics, School of Biosciences, University of Nottingham, Sutton Bonington Campus, Loughborough LE12 5RD, United Kingdom*

*^c^National Centre for Macromolecular Hydrodynamics, School of Biosciences, University of Nottingham, Sutton Bonington Campus, Loughborough LE12 5RD, United Kingdom*

*^d^Biomaterials Laboratory, School of Biosciences, University of Nottingham, Sutton Bonington Campus, Loughborough LE12 5RD, United Kingdom*

*Correspondence to Ian Fisk, [ian.fisk@nottingham.ac.uk](mailto:ian.fisk@nottingham.ac.uk)

***Table 1: CATA (orthonasal aroma) and TCATA (retronasal flavour, taste and mouthfeel) attributes and definitions provided to consumers during familiarisation session.***

|  | **Attributes** | **Definition** |
| --- | --- | --- |
| **Aroma** | *Fruity* | Smell of fruits such as banana, green apple, pineapple, peach, lemon, lime, orange or grapefruit. |
|  | *Malty* | Small of cereals or grains. Can be related to smell of Ovaltine drink. |
|  | *Hoppy* | Smell of hops, which can be floral/herbal. |
|  | *Stale* | Musty smell or smell of wet paper/cardboard. |
|  | *Cooked Vegetable* | Smell of cooked vegetables such as cabbage or sweetcorn. Can also be related to a sulphur smell. |
|  | *Alcohol* | Smell of alcohol/spirits. |
| **Flavour and Taste** | *Malty Flavour* | Flavour of malty cereals. Can be related to smell of Ovaltine drink. |
|  | *Hoppy Flavour* | Flavour of hops which can be flowery and herbal. |
|  | *Fruity Flavour* | Flavour of fruit characteristics – including banana, apple, pineapple, peach, lemon, orange. |
|  | *Bitter Taste* | Taste stimulated by strong black coffee, beer, red wine or tonic water. |
|  | *Sweet Taste* | Taste stimulated by sugar when experienced in mouth. |
|  | *Sour Taste* | Taste stimulated by acids when experienced in mouth. |
| **Mouthfeel** | *Fullness/Body* | Feeling of thickness/fullness as beer is moved around in the mouth. |
|  | *Alcohol Warming Sensation* | The feeling of warming which is characteristic of ethanol throughout the mouth. |
|  | *Tingly Sensation* | Perception of irritation such as prickling, stinging and bubbles bursting in mouth from carbonation. The feeling of pins and needles. |
|  | *Astringent Mouthfeel* | The feeling in mouth of roughing, puckering and drying. |

***Table 2: Effect of ethanol and α-amylase*** ***on a) lager style beer; b) stout style beer. Values in bold are significant at p<0.05. ᵃᵇᶜᵈDifferent letters within a column represent significant differences among samples.***

| ***A - Lager*** | *Furfural* | *Ethyl Acetate* | *3-Methylbutanal* | *Isoamyl Alcohol* | *Phenylethyl Alcohol* | *Hexanal* | *Isoamyl Acetate* | *Ethyl Hexanoate* | *Linalool* |
| --- | --- | --- | --- | --- | --- | --- | --- | --- | --- |
| 0% Ethanol | 6.74E+07ᵃ | 2.79E+09ᵃ | 3.98E+08ᵃ | 2.24E+09ᵃ | 2.69E+08ᵃ | 1.55E+08ᵃ | 2.79E+09ᵃ | 5.46E+08ᵃ | 6.36E+07ᵃ |
| 0% Ethanol + α-amylase | 5.86E+07ᵃ | 2.97E+09ᵃ | 3.16E+08ᵇ | 2.10E+09ᵃ | 2.32E+08ᵃᵇ | 1.05E+08ᵇ | 1.97E+09ᶜ | 2.17E+08ᶜ | 2.47E+07ᵇ |
| 5% Ethanol | 4.25E+07ᵃ | 1.33E+09ᵇ | 1.92E+08ᶜ | 1.44E+09ᵇ | 2.18E+08ᵃᵇ | 9.79E+07ᵇᶜ | 2.30E+09ᵇ | 4.39E+08ᵇ | 4.54E+07ᵃᵇ |
| 5% Ethanol + α-amylase | 3.91E+07ᵃ | 1.32E+09ᵇ | 1.42E+08ᵈ | 1.36E+09ᵇ | 1.69E+08ᵇ | 7.34E+07ᶜ | 1.72E+09d | 1.97E+08ᶜ | 2.31E+07ᵇ |
| p values | 0.059 | **< 0.0001** | **< 0.0001** | **< 0.0001** | **0.014** | **< 0.0001** | **< 0.0001** | **< 0.0001** | **0.004** |

| ***B - Stout*** | *Furfural* | *Ethyl Acetate* | *3-Methylbutanal* | *Isoamyl Alcohol* | *Phenylethyl Alcohol* | *Hexanal* | *Isoamyl Acetate* | *Ethyl Hexanoate* | *Linalool* |
| --- | --- | --- | --- | --- | --- | --- | --- | --- | --- |
| 0% Ethanol | 5.00E+07ᵃ | 1.02E+09ᵃ | 5.09E+08ᵃ | 1.65E+09ᵃ | 1.51E+08ᵃ | 1.79E+08ᵃ | 8.10E+08ᵃ | 4.39E+08ᵃ | 7.40E+07ᵃ |
| 0% Ethanol + α-amylase | 5.61E+07ᵃ | 1.10E+09ᵃ | 4.27E+08ᵇ | 1.61E+09ᵃ | 1.26E+08ᵃ | 1.05E+08ᵇ | 5.52E+08ᵇ | 1.82E+08ᶜ | 3.17E+07ᶜ |
| 5% Ethanol | 3.20E+07ᵇ | 3.69E+08ᵇ | 2.22E+08ᶜ | 9.53E+08ᵇ | 1.26E+08ᵃ | 1.07E+08ᵇ | 4.84E+08ᵇᶜ | 2.93E+08ᵇ | 4.80E+07ᵇ |
| 5% Ethanol + α-amylase | 3.26E+07ᵇ | 3.77E+08ᵇ | 1.74E+08ᵈ | 8.98E+08ᵇ | 1.12E+08ᵃ | 6.03E+07ᵇ | 3.55E+08ᶜ | 1.33E+08ᶜ | 2.56E+07ᶜ |
| p values | **0.000** | **< 0.0001** | **< 0.0001** | **< 0.0001** | 0.145 | **0.000** | **< 0.0001** | **< 0.0001** | **< 0.0001** |

***Figure 1: Correlation plot summarising the relationship between saliva, ethanol and corresponding logP values across samples. Red denotes a strong positive relationship and blue denotes a strong negative relationship.***
